# 2,2′-(2,6-Pyridinedi­yl)diquinoline

**DOI:** 10.1107/S1600536810006033

**Published:** 2010-02-20

**Authors:** Luis Ángel Garza Rodríguez, Sylvain Bernès, Perla Elizondo Martínez, Blanca Nájera Martínez

**Affiliations:** a Laboratorio de Química Industrial, CELAES, Facultad de Ciencias Químicas, UANL, Pedro de Alba S/N, 66451 San Nicolás de los Garza, N.L., Mexico; b DEP Facultad de Ciencias Químicas, UANL, Guerrero y Progreso S/N, Col. Treviño, 64570 Monterrey, N.L., Mexico

## Abstract

The title mol­ecule, C_23_H_15_N_3_, is a terpyridine derivative resulting from the Friedländer annulation between 2,6-diacetyl­pyridine and *N*,*N′*-bis­(2-amino­benz­yl)ethyl­ene­di­amine. The asymmetric unit contains one half-mol­ecule, the complete mol­ecule being generated by a mirror plane (one N atom and one C atom lie on the plane). The mol­ecule, although aromatic, is deformed from planarity as a result of crystal packing forces: mol­ecules are stacked along the short *c* axis, with a short separation of 3.605 (1) Å between the mean planes. The bent mol­ecular shape is reflected in the dihedral angle of 16.10 (5)° between the essentially planar quinoline groups. In addition to π⋯π inter­actions, the crystal structure features weak inter-stack C—H⋯N contacts involving atoms of the central pyridine rings which lie in a common crystallographic *m* plane.

## Related literature

For the synthesis and the coordination behavior of the title mol­ecule, see: Bertrand *et al.* (2009 ▶); Harris *et al.* (1969 ▶); Klassen *et al.* (1975 ▶). For a terpyridine derivative closely related to the title mol­ecule, see: Sasaki *et al.* (1998 ▶). For the Friedländer condensation as a tool for the preparation of quinolines, see: Da Costa *et al.* (2009 ▶); Sridharan *et al.* (2009 ▶).
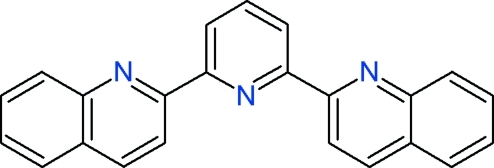


## Experimental

### 

#### Crystal data

C_23_H_15_N_3_
*M* *_r_* = 333.38
Orthorhombic, 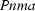

*a* = 11.960 (2) Å
*b* = 34.509 (6) Å
*c* = 3.9509 (5) Å
*V* = 1630.7 (5) Å^3^
*Z* = 4
Mo *K*α radiation
μ = 0.08 mm^−1^
*T* = 298 K
0.40 × 0.20 × 0.10 mm

#### Data collection

Siemens P4 diffractometer
5603 measured reflections
1469 independent reflections
1032 reflections with *I* > 2σ(*I*)
*R*_int_ = 0.031
2 standard reflections every 48 reflections  intensity decay: 1%

#### Refinement

*R*[*F* ^2^ > 2σ(*F* ^2^)] = 0.041
*wR*(*F* ^2^) = 0.118
*S* = 1.02
1469 reflections
122 parameters
H-atom parameters constrained
Δρ_max_ = 0.16 e Å^−3^
Δρ_min_ = −0.11 e Å^−3^

### 

Data collection: *XSCANS* (Siemens, 1996 ▶); cell refinement: *XSCANS*; data reduction: *XSCANS*; program(s) used to solve structure: *SHELXTL-Plus* (Sheldrick, 2008 ▶); program(s) used to refine structure: *SHELXTL-Plus*; molecular graphics: *SHELXTL-Plus* and *Mercury* (Macrae *et al.*, 2008 ▶); software used to prepare material for publication: *SHELXTL-Plus*.

## Supplementary Material

Crystal structure: contains datablocks I, global. DOI: 10.1107/S1600536810006033/xu2726sup1.cif

Structure factors: contains datablocks I. DOI: 10.1107/S1600536810006033/xu2726Isup2.hkl

Additional supplementary materials:  crystallographic information; 3D view; checkCIF report

## Figures and Tables

**Table 1:** Hydrogen-bond geometry (Å, °)

*D*—H⋯*A*	*D*—H	H⋯*A*	*D*⋯*A*	*D*—H⋯*A*
C1—H1*A*⋯N1^i^	0.93	2.72	3.641 (3)	169

Symmetry code: (i) 
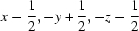
.

**Fig. 1.**
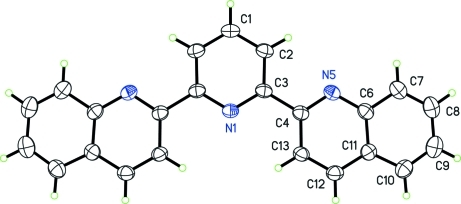
The structure of the title compound, with displacement ellipsoids at the 30% probability level for non-H atoms. Non-labeled atoms are generated by symmetry code x, 1/2-y, z.

**Fig. 2.**
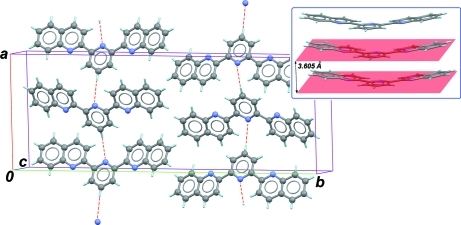
A part of the crystal structure, viewed down c axis. Dashed lines represent non-bonding intermolecular contacts. The inset shows a part of a stack along [001]. Two least-squares planes are represented (red), which were computed using all atoms in each selected molecule (Macrae et al., 2008).

## supplementary crystallographic information

### Comment

Thirty years after cisplatin was approved by the FDA for its use as a chemotherapy drug, studies regarding interactions between platinum-based complexes and basic sites in DNA remain actives. Recently, Bertrand *et al.* (2009) showed that Pt^II^ cationic complexes bearing 2,2':6',2"-terpyridine or a terpyridine derivative as ligand have the ability to platinate the human telomeric G-quadruplex. Interestingly, both the binding affinity and the platination activity seem to be determined by the extension of the aromatic surface of the terpyridine derivative. One of the ligands used in that work was 2,2'-(2,6-pyridinediyl)bis-quinoline, synthesized through the Friedländer condensation (Da Costa *et al.*, 2009; Sridharan *et al.*, 2009) between 2,6-diacetylpyridine and 2-nitrobenzaldehyde. We now report the crystal structure of this aromatic ligand.

The title terpyridine derivative was obtained as a by-product during the preparation of a macrocyclic ligand (see *Experimental*). More suitable synthesis are however available in the literature (Harris *et al.*, 1969; Klassen *et al.*, 1975; Bertrand *et al.*, 2009). The molecule (Fig. 1) displays the crystallographic *m* symmetry, with atoms N1, C1 and H1A placed in the mirror planes normal to [010]. The molecular conformation observed in the solid-state is not suitable for coordination through the three N atoms: the quinoline N atoms are placed in a *trans* arrangement with respect to the central pyridine N atom, while a *cis*,*cis* conformation is required for the molecule to be a terdentate ligand. However, as invariably found in non-hindered terpyridine derivatives, aromatic fragments are free to rotate, for example about the C3—C4 bond in the case of the title molecule. Such a behavior has been reported, for example, for the coordination to Ru^II^ of a closely related terpyridine ligand, namely 2,6-bis(5,6,7,8-tetrahydroquinol-2-yl)pyridine (Sasaki *et al.*, 1998).

Molecules are stacked along the short axis *c*, at a distance of 3.605 Å (separation between two mean planes passing through two neighboring molecules in the [001] direction, see Fig.2, inset). This short separation, although larger than that observed in graphite (*ca*. 3.36 Å), results in strong π···π interactions in the stacks, which, in turn, deform the molecules from planarity. The dihedral angle between the central pyridine ring and the quinoline substituent is 8.13 (8)°. The bent shape is also reflected in the dihedral angle between quinoline systems, 16.10 (5)° (Fig. 2, inset). Finally, the crystal structure is completed by weak intermolecular C—H···N contacts (Table 1 and Fig. 2), linking the stacks in the [100] direction.

### Experimental

A mixture of 305 mg of 2,6-diacetylpyridine and 823 mg of Ce(NO_3_)_3_.6H_2_O in methanol (25 ml) was refluxed for 30 min, followed by slow addition of a dissolution of *N*,*N'*-bis(2-aminobenzyl)ethylenediamine (530 mg in 25 ml methanol). The mixture was kept under these conditions for 3.5 h, and then cooled to room temperature, giving a red precipitate. After 1.5 month, the resulting solid was filtered, washed with cold methanol, diethyl ether, and air dried. Suitable single crystals were picked off from the solid. m.p. 495–497 K (lit. 500–501 K: Klassen *et al.*, 1975).

### Refinement

All H atoms were placed in idealized positions, with C—H bond lengths fixed to 0.93 Å. Isotropic displacement parameters for H atoms were calculated from displacements of parent C atoms: *U*_iso_(H) = 1.2*U*_eq_(C).

### Crystal data

**Table:**

C_23_H_15_N_3_	*D*_x_ = 1.358 Mg m^−^^3^
*M**_r_* = 333.38	Melting point = 495–497 K
Orthorhombic, *P**n**m**a*	Mo *K*α radiation, λ = 0.71073 Å
Hall symbol: -P 2ac 2n	Cell parameters from 100 reflections
*a* = 11.960 (2) Å	θ = 5.5–11.7°
*b* = 34.509 (6) Å	µ = 0.08 mm^−^^1^
*c* = 3.9509 (5) Å	*T* = 298 K
*V* = 1630.7 (5) Å^3^	Plate, orange
*Z* = 4	0.40 × 0.20 × 0.10 mm
*F*(000) = 696	

### Data collection

**Table:**

Siemens P4 diffractometer	*R*_int_ = 0.031
Radiation source: X-ray	θ_max_ = 25.1°, θ_min_ = 2.4°
graphite	*h* = −14→14
ω scans	*k* = −41→41
5603 measured reflections	*l* = −4→4
1469 independent reflections	2 standard reflections every 48 reflections
1032 reflections with *I* > 2σ(*I*)	intensity decay: 1%

### Refinement

**Table:**

Refinement on *F*^2^	Secondary atom site location: difference Fourier map
Least-squares matrix: full	Hydrogen site location: inferred from neighbouring sites
*R*[*F*^2^ > 2σ(*F*^2^)] = 0.041	H-atom parameters constrained
*wR*(*F*^2^) = 0.118	*w* = 1/[σ^2^(*F*_o_^2^) + (0.0614*P*)^2^ + 0.1644*P*] where *P* = (*F*_o_^2^ + 2*F*_c_^2^)/3
*S* = 1.01	(Δ/σ)_max_ < 0.001
1469 reflections	Δρ_max_ = 0.16 e Å^−^^3^
122 parameters	Δρ_min_ = −0.11 e Å^−^^3^
0 restraints	Extinction correction: *SHELXTL-Plus*, Fc^*^=kFc[1+0.001xFc^2^λ^3^/sin(2θ)]^-1/4^
0 constraints	Extinction coefficient: 0.0069 (16)
Primary atom site location: structure-invariant direct methods	

### Fractional atomic coordinates and isotropic or equivalent isotropic displacement parameters (Å^2^)

**Table:**

	*x*	*y*	*z*	*U*_iso_*/*U*_eq_	
N1	0.05056 (13)	0.2500	0.0324 (4)	0.0465 (5)	
C1	−0.15745 (18)	0.2500	−0.2714 (6)	0.0572 (6)	
H1A	−0.2280	0.2500	−0.3703	0.069*	
C2	−0.10509 (12)	0.28415 (5)	−0.1980 (4)	0.0539 (4)	
H2A	−0.1388	0.3077	−0.2506	0.065*	
C3	−0.00108 (11)	0.28327 (4)	−0.0443 (4)	0.0465 (4)	
C4	0.05844 (12)	0.32017 (4)	0.0278 (4)	0.0468 (4)	
N5	0.00977 (10)	0.35190 (4)	−0.0808 (3)	0.0522 (4)	
C6	0.06408 (13)	0.38627 (4)	−0.0411 (4)	0.0522 (4)	
C7	0.01369 (16)	0.42018 (5)	−0.1667 (5)	0.0667 (5)	
H7A	−0.0563	0.4188	−0.2686	0.080*	
C8	0.06583 (19)	0.45476 (5)	−0.1410 (5)	0.0752 (6)	
H8A	0.0319	0.4769	−0.2279	0.090*	
C9	0.17008 (19)	0.45757 (5)	0.0147 (5)	0.0762 (6)	
H9A	0.2049	0.4816	0.0326	0.091*	
C10	0.22110 (16)	0.42554 (5)	0.1402 (5)	0.0660 (5)	
H10A	0.2907	0.4278	0.2437	0.079*	
C11	0.16971 (13)	0.38897 (4)	0.1153 (4)	0.0528 (4)	
C12	0.21698 (13)	0.35438 (4)	0.2379 (4)	0.0555 (4)	
H12A	0.2859	0.3549	0.3468	0.067*	
C13	0.16215 (12)	0.32044 (4)	0.1973 (4)	0.0511 (4)	
H13A	0.1925	0.2975	0.2804	0.061*	

### Atomic displacement parameters (Å^2^)

**Table:**

	*U*^11^	*U*^22^	*U*^33^	*U*^12^	*U*^13^	*U*^23^
N1	0.0355 (9)	0.0555 (11)	0.0484 (10)	0.000	0.0022 (8)	0.000
C1	0.0364 (11)	0.0734 (16)	0.0619 (14)	0.000	−0.0071 (11)	0.000
C2	0.0404 (8)	0.0637 (10)	0.0576 (10)	0.0072 (7)	−0.0014 (7)	−0.0021 (8)
C3	0.0361 (7)	0.0586 (9)	0.0448 (8)	0.0043 (7)	0.0045 (6)	−0.0018 (7)
C4	0.0395 (8)	0.0554 (9)	0.0456 (8)	0.0054 (7)	0.0048 (7)	−0.0030 (7)
N5	0.0442 (7)	0.0569 (8)	0.0555 (8)	0.0078 (6)	0.0016 (6)	−0.0002 (7)
C6	0.0506 (9)	0.0563 (10)	0.0498 (9)	0.0089 (8)	0.0066 (7)	−0.0027 (8)
C7	0.0688 (11)	0.0654 (11)	0.0659 (11)	0.0147 (9)	0.0013 (9)	0.0016 (9)
C8	0.0985 (16)	0.0599 (12)	0.0672 (12)	0.0145 (11)	0.0066 (12)	0.0053 (10)
C9	0.0981 (16)	0.0617 (11)	0.0687 (13)	−0.0086 (11)	0.0121 (11)	−0.0025 (10)
C10	0.0672 (11)	0.0673 (11)	0.0635 (11)	−0.0064 (10)	0.0044 (9)	−0.0083 (9)
C11	0.0522 (9)	0.0561 (10)	0.0501 (9)	0.0020 (7)	0.0060 (8)	−0.0062 (8)
C12	0.0443 (8)	0.0648 (11)	0.0575 (10)	0.0031 (8)	−0.0032 (7)	−0.0078 (8)
C13	0.0426 (8)	0.0545 (9)	0.0561 (9)	0.0077 (7)	−0.0033 (7)	−0.0043 (7)

### Geometric parameters (Å, °)

**Table:**

N1—C3^i^	1.3383 (16)	C7—C8	1.350 (2)
N1—C3	1.3383 (16)	C7—H7A	0.9300
C1—C2	1.3658 (18)	C8—C9	1.394 (3)
C1—C2^i^	1.3658 (18)	C8—H8A	0.9300
C1—H1A	0.9300	C9—C10	1.357 (2)
C2—C3	1.385 (2)	C9—H9A	0.9300
C2—H2A	0.9300	C10—C11	1.407 (2)
C3—C4	1.486 (2)	C10—H10A	0.9300
C4—N5	1.3123 (18)	C11—C12	1.407 (2)
C4—C13	1.410 (2)	C12—C13	1.352 (2)
N5—C6	1.3615 (19)	C12—H12A	0.9300
C6—C7	1.407 (2)	C13—H13A	0.9300
C6—C11	1.410 (2)		
			
C3^i^—N1—C3	118.13 (17)	C6—C7—H7A	119.6
C2—C1—C2^i^	119.3 (2)	C7—C8—C9	120.52 (18)
C2—C1—H1A	120.3	C7—C8—H8A	119.7
C2^i^—C1—H1A	120.3	C9—C8—H8A	119.7
C1—C2—C3	119.07 (15)	C10—C9—C8	120.48 (18)
C1—C2—H2A	120.5	C10—C9—H9A	119.8
C3—C2—H2A	120.5	C8—C9—H9A	119.8
N1—C3—C2	122.20 (14)	C9—C10—C11	120.57 (18)
N1—C3—C4	118.07 (13)	C9—C10—H10A	119.7
C2—C3—C4	119.69 (13)	C11—C10—H10A	119.7
N5—C4—C13	122.69 (14)	C12—C11—C10	124.15 (16)
N5—C4—C3	116.09 (13)	C12—C11—C6	117.07 (14)
C13—C4—C3	121.21 (13)	C10—C11—C6	118.78 (15)
C4—N5—C6	118.54 (13)	C13—C12—C11	119.96 (14)
N5—C6—C7	118.67 (15)	C13—C12—H12A	120.0
N5—C6—C11	122.39 (14)	C11—C12—H12A	120.0
C7—C6—C11	118.92 (15)	C12—C13—C4	119.27 (14)
C8—C7—C6	120.72 (18)	C12—C13—H13A	120.4
C8—C7—H7A	119.6	C4—C13—H13A	120.4
			
C2^i^—C1—C2—C3	−1.4 (3)	C6—C7—C8—C9	−0.8 (3)
C3^i^—N1—C3—C2	0.1 (3)	C7—C8—C9—C10	0.5 (3)
C3^i^—N1—C3—C4	−177.46 (10)	C8—C9—C10—C11	0.0 (3)
C1—C2—C3—N1	0.6 (3)	C9—C10—C11—C12	179.83 (16)
C1—C2—C3—C4	178.17 (16)	C9—C10—C11—C6	−0.3 (3)
N1—C3—C4—N5	173.63 (14)	N5—C6—C11—C12	−1.3 (2)
C2—C3—C4—N5	−4.0 (2)	C7—C6—C11—C12	179.88 (15)
N1—C3—C4—C13	−5.2 (2)	N5—C6—C11—C10	178.75 (14)
C2—C3—C4—C13	177.17 (14)	C7—C6—C11—C10	0.0 (2)
C13—C4—N5—C6	3.0 (2)	C10—C11—C12—C13	−178.88 (16)
C3—C4—N5—C6	−175.81 (13)	C6—C11—C12—C13	1.2 (2)
C4—N5—C6—C7	178.05 (14)	C11—C12—C13—C4	0.8 (2)
C4—N5—C6—C11	−0.7 (2)	N5—C4—C13—C12	−3.1 (2)
N5—C6—C7—C8	−178.24 (16)	C3—C4—C13—C12	175.65 (14)
C11—C6—C7—C8	0.6 (2)		

Symmetry codes: (i) *x*, −*y*+1/2, *z*.

### Hydrogen-bond geometry (Å, °)

**Table:**

*D*—H···*A*	*D*—H	H···*A*	*D*···*A*	*D*—H···*A*
C1—H1A···N1^ii^	0.93	2.72	3.641 (3)	169

Symmetry codes: (ii) *x*−1/2, −*y*+1/2, −*z*−1/2.
